# Molecular evidence of *Bartonella* spp. in wild felids from Brazil

**DOI:** 10.1007/s11259-026-11579-5

**Published:** 2026-10-17

**Authors:** Clara Morato Dias, Daniel Antônio Braga Lee, João Vitor dos Santos Alves da Silva, Laura Berger Nunes, Joares Adenilson May Júnior, Vinícius Baggio-Souza, Felipe Bortolotto Peters, Marina Ochoa Favarini, Ana Paula Neuschrank Albano, Suelen Dias Segui, Silvia Resende Terra, Flávia Pereira Tirelli, Jorge Cherem, Hugo Borghesan Morzele, Marcos Adriano Tortato, Adriano Rodrigues Lagos, Felipe Viana Manzano, Tiago Chagas de Oliveira Tourinho, Rosangela Zacarias Machado, João Fabio Soares, Marcos Rogério André

**Affiliations:** 1 Vector-Borne Bioagents Laboratory, Departamento de Patologia, Reprodução e Saúde Única, Faculdade de Ciências Agrárias e Veterinárias, Universidade Estadual “Júlio de Mesquita Filho” - FCAV/UNESP, Jaboticabal, SP Brasil; 2 Laboratório de Protozoologia e Rickettsioses Vetoriais (ProtozooVet), Faculdade de Veterinária, Universidade Federal do Rio Grande do Sul (UFRGS), Porto Alegre, RS Brazil; 3 Panthera Brasil, Poconé, MT Brazil; 4 Centro Universitário Univinte, Capivari de Baixo, SC Brazil; 5 Ampara Silvestres, Poconé, MT Brazil; 6 Instituto Pro-Carnívoros, Atibaia, SP Brazil; 7 Bird and Mammal Evolution, Systematics and Ecology Lab, Departamento de Zoologia, Instituto de Biociências, Universidade Federal do Rio Grande do Sul, Porto Alegre, RS Brazil; 8 Hospital de Clínica Veterinária, Universidade Federal de Pelotas, Pelotas, RS Brasil; 9 Bióloga, Candiota, RS Brazil; 10 Instituto de Ciências Básicas da Saúde, Universidade Federal do Rio Grande do Sul (UFRGS), Porto Alegre, RS Brazil; 11 Tapir consultoria Ambiental LTDA, Gravatal, SC Brazil; 12 Axia Energia, Rio de Janeiro, RJ Brazil; 13 Vector-Borne Bioagents Laboratory (VBBL), Department of Pathology, Reproduction and One Health, Faculty of Agrarian and Veterinary Sciences Júlio de Mesquita Filho, (UNESP), Jaboticabal Campus, Via Prof. Access Paulo Donato Castellane, s/n, Rural Zone, CEP: 14884-900 Jaboticabal, São Paulo Brazil

**Keywords:** *Bartonella* species, *Herpailurus yagouaroundi*, *Leopardus geoffroyi*, *Leopardus pardalis*, *Leopardus wiedii*, *Panthera onca*, Wildlife

## Abstract

Among Felidae, domestic cats play a role as reservoirs for *Bartonella henselae*, *Bartonella clarridgeiae*, and *Bartonella koehlerae*. Despite the well-known relationship between *Bartonella* spp. and domestic cats, to date, there is still a paucity of reports on *Bartonella* spp. in wild felids. The aim of this study was to molecularly detect and characterize *Bartonella* spp. in blood and tissue samples from wild felids sampled in Brazil. Among 291 DNA samples (211 blood, 77 spleens and three livers), 3.09% (9/291) were positive in the Quantitative Real-Time PCR (qPCR) assay for *Bartonella* spp. based on the *nuoG* gene: 1/44 *Leopardus pardalis*, 3/61 *Leopardus geoffroyi*, 1/66 *Panthera onca*, 1/3 *Herpailurus yagouaroundi*, and 3/32 *Leopardus wiedii*. In the phylogenetic analyses, while the *gltA* sequence detected in *L. wiedii* grouped with *Bartonella henselae*, the *gltA* and *ftsZ* sequences detected in *L. geoffroyi* clustered within the same subclade as *Bartonella* sp. sequences previously identified in wild rodents and associated fleas. These findings reinforce that besides harboring *Bartonella* species associated with cats, wild felids also may act as accidental hosts for *Bartonella* species associated with wild small mammals, possibly as a consequence of predation.

**Supplementary Information:**

The online version contains supplementary material available at https://doi.org/10.1007/s11259-026-11579-5.

## Introduction

The genus *Bartonella* comprises fastidious, facultative intracellular, Gram-negative α-proteobacteria that parasitize erythrocytes and endothelial cells of several mammalian species (Kosoy et al. 2012). These bacteria usually exhibit high host specificity, often establishing long-lasting asymptomatic bacteremia in their natural mammalian reservoirs. In humans, incidental infections can result in a range of clinical manifestations, most commonly cat-scratch disease (CSD), while other, less frequent manifestations include bacillary angiomatosis and endocarditis (Kosoy et al. 2012).

Among felid species, domestic cats play a role as reservoirs for *Bartonella henselae*, *Bartonella clarridgeiae*, and *Bartonella koehlerae* (Kosoy et al. 2012). Wild felids can also harbor *Bartonella* species found in domestic cats, such as *B. henselae* and *B. koehlerae*, as well as distinct *Bartonella* lineages (Molia et al. 2004, 2016). However, the real role of wild felids as reservoirs for these agents remains a topic of ongoing debate (Obiegala et al. 2024). Different research groups have molecularly detected *Bartonella* spp. in wild felids from Japan (Tateno et al. 2013), Germany (Obiegala et al. 2024), South Africa, Kenya, Tanzania, Namibia (Molia et al. 2004), the United States of America (Boulouis et al. 2020), Mexico (López-Pérez et al. 2017), and Brazil (Guimarães et al. 2010; Filoni et al. 2012; Souza et al. 2021). In Brazil, while *B. henselae* DNA was detected in *Leopardus geoffroyi*, *L. tigrinus*, *L. wiedii*, *L. pardalis*, *Panthera onca*, and *Herpailurus yagouaroundi*, *B. koehlerae* was detected only in *L. wiedii* (Guimarães et al. 2010; Filoni et al. 2012; Souza et al. 2021).

Out of eleven wild felid species recognized in Brazil, eight can be found in the state of Rio Grande do Sul (RS), southern Brazil (Souza et al. 2021). Despite the country’s ecological diversity and rich wild feline fauna, few studies have explored the molecular diversity of *Bartonella* spp. in these hosts. Therefore, this study aimed to molecularly detect and characterize *Bartonella* spp. in blood, liver, and spleen samples from wild felids across the North, Northeast, Central-West, Southeast, and South regions of Brazil, contributing to a better understanding of their circulation and potential zoonotic risks.

## Materials and methods

### Sampling and studied area

**Fig. 1:**
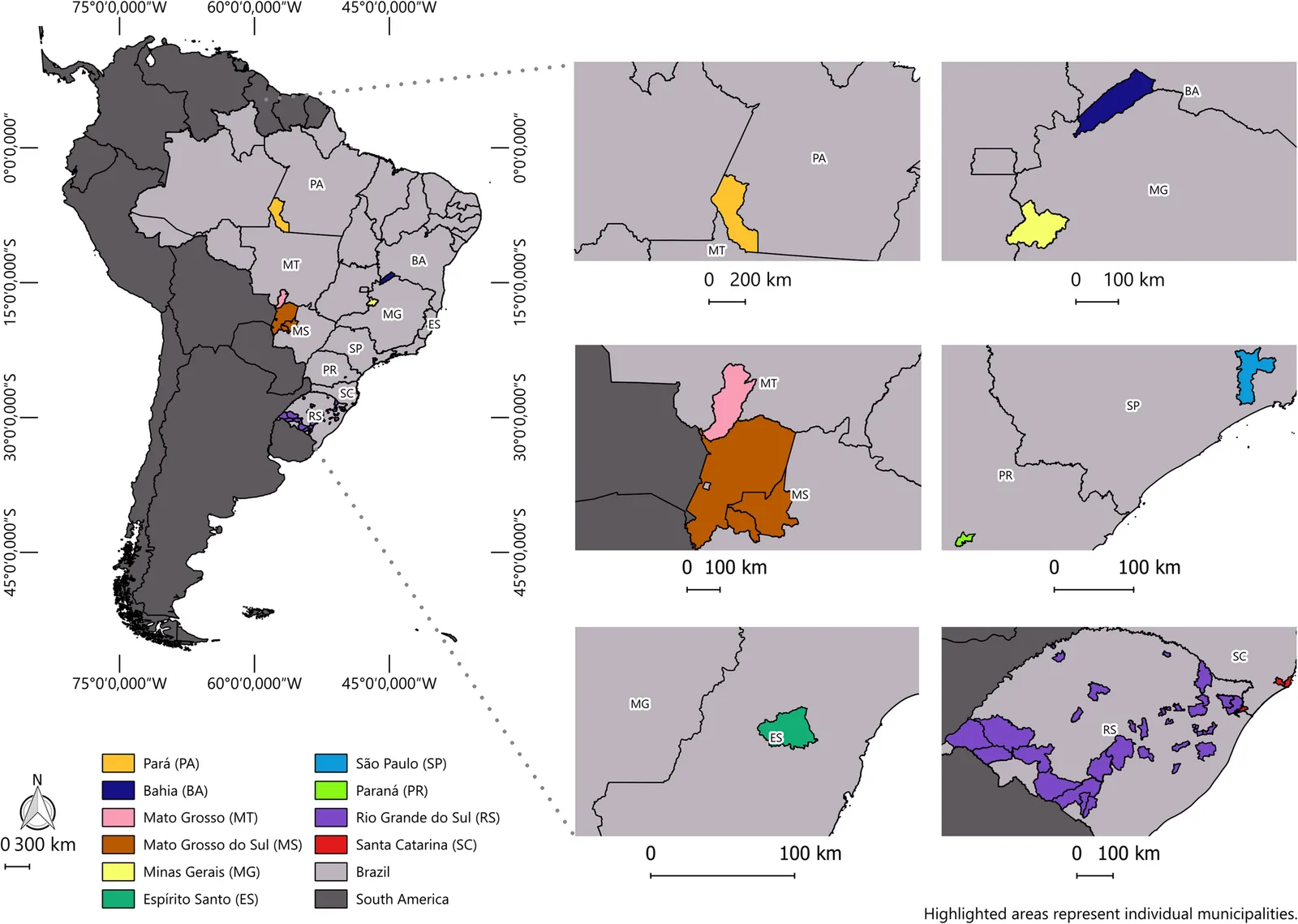
Map generated by QGIS 2.18.2.8, showing the states of Brazil and the location of cities by state where wild felids were sampled in the present study in different colors

Between 2017 and 2026, a total of 211 blood, 77 spleen and three liver samples were collected from wild felids across nine Brazilian states, covering the North, Northeast, Central-West, Southeast, and South regions of the country. Sampling was conducted in 51 municipalities, with the sampling area shown in Fig. 1 and the detailed distribution of samples by animal species provided in Supplementary Material 3.

The taxonomic identification of the wild felids followed the guidelines of the Taxonomy Committee of the Brazilian Society of Mammalogy (CT-SBMz) (Abreu et al. 2025). Felids were captured using different methods according to their body size. While *H. yagouaroundi*, *L. wiedii*, *L. pardalis* and *L. geoffroyi* were captured using Tomahawk live traps, *Panthera onca* individuals were captured using foot snares (de Araujo et al. 2021). Capture devices were placed in areas with previous reports of sightings, camera traps, and evidence of scat and/or footprints of wild felines. The traps were available in the field for the period of 7–15 days and were monitored at least once a day for capture occasions. After capture, the animals were restrained with a combination of ketamine (approximately 10 mg/kg) and xylazine (approximately 1 mg/kg) intramuscularly (Souza et al. 2021). Approximately 2 mL of blood was collected from each animal into EDTA-tubes (Sigma-Aldrich, St. Louis, Missouri, USA) by jugular and/or cephalic vein puncture under aseptic conditions. Immediately after collection, the blood samples were kept refrigerated in reusable ice packs and subsequently transported to a field base, where they were stored at − 20 °C until further molecular analyses.

### DNA extraction

DNA was extracted from 291 wild felids’ biological samples (211 blood, 77 spleen, and three liver samples) using a commercial extraction kit PureLink™ Genomic DNA Kit (Invitrogen, Carlsbad, California, USA), following the manufacturer’s instructions. After extraction, DNA concentration was quantified by spectrophotometry using a NanoDrop™ (Thermo Fisher Scientific, Waltham, Massachusetts, USA). The DNA concentrations of the samples were standardized to approximately 100 ng/µL. The DNA samples were stored at − 20 °C until further molecular analyses.

### Molecular screening and characterization of *Bartonella* spp

DNA samples were subjected to a qPCR to detect and quantify the number of copies of a fragment of the *Bartonella* nicotinamide adenine dinucleotide dehydrogenase subunit gamma (*nuoG*) gene per microliter (André et al. 2015). DNA samples were evaluated in duplicates. When differences in Cq values were greater than 0.5, the samples were retested in triplicates. In the construction of the standard curve of each reaction, serial dilutions were performed at different concentrations (1.0 × 10^7^ to 1.0 × 10^0^ copies) of a plasmid encoding an 83 bp fragment of the *nuoG* gene of *B. henselae* (pIDTSMART; Integrated DNA Technologies, Coralville, Iowa, United States), which was also used as positive controls. The number of plasmid copies was determined by the formula (XG/µL DNA/ [Plasmid Length (BP) × 660]) × 6. 22 × 10^23^ × plasmid copies/µL. The amplification efficiency (E) was calculated according to the slope of the standard curve using the formula E = 10^− 1/slope^ (Bustin et al. 2009). UltraPure™ DNase/RNase-Free Distilled Water (Thermo Fisher Scientific, Waltham, MA, USA) was used as a negative control for each reaction performed. The qPCR assays were carried out in a C1000 Touch™ Thermal Cycler with CFX96™ Real-Time PCR Detection System (Bio-Rad Laboratories, Hercules, CA, USA).

Samples positive in the qPCR were subjected to PCR assays targeting eigth molecular markers, selected based on previously published protocols widely used for the molecular characterization and phylogenetic analysis of *Bartonella* spp., namely *gltA* (citrate synthase) (Norman et al. 1995), *ftsZ* (cell division protein FtsZ) (Paziewska et al. 2011), *groEL* (60 kDa chaperonin GroEL) (Paziewska et al. 2011), *nuoG* (NADH dehydrogenase I subunit G) (Colborn et al. 2010), *pap31* (31-kDa heme-binding protein) (Maggi and Breitschwerdt 2005a), *rpoB* (DNA-directed RNA polymerase subunit beta) (Renesto et al. 2001), *ribC* (riboflavin synthase alpha chain) (Johnson et al. 2003), and 16–23 S rRNA intergenic spacer region (Maggi and Breitschwerdt 2005b) (Supplementary material 1).

### DNA purification, sequencing and phylogenetic analysis

Amplicons obtained in the conventional PCR assays were purified by using ExoSAP-IT™ PCR Product Cleanup Reagent (Thermo Fisher Scientific, Waltham, MA, USA) and submitted to Sanger sequencing (Sanger et al. 1977) in both directions (forward and reverse) at the Centro de Estudos do Genoma Humano e Células Tronco (University of São Paulo, São Paulo, Brazil) using the BigDye™ Terminator v3.1 Cycle Sequencing Kit (Thermo Fisher Scientific, Waltham, MA, USA).

The received sequences were visualized, trimmed, and a consensus sequence was assembled using the softwares BioEdit 7.7 (Hall 1999), Phred/Phrap and Consed (Ewing et al. 1998). All consensus sequences were submitted to BLASTn analysis (https://blast.ncbi.nlm.nih.gov/Blast.cgi*)* to check the percentage of identity with sequences deposited previously in the GenBank database. The alignments were performed using the software MAFFT 7.0 (Katoh and Standley 2013). For each alignment, the best-fitting evolutionary model was chosen by using the jModeltest v.2.1.10 (Darriba et al. 2012) software through the PAUP4* Version 4c software (Swofford 2003).

A Maximum Likelihood (ML) analysis was performed using the IQ-TREE2 software (Nguyen and Haeseler 2016) using 10^3^ Ultra-Fast Bootstrap Replicates for each alignment. The resulting phylogenetic trees were visualized and edited using the FigTree 1.4.4 (https://tree.bio.ed.ac.uk/software/figtree/*)* software.

## Results

Quantitative Real-Time PCR assays for *Bartonella* spp. based on the *nuoG* gene detected a positivity of 3.09% (9/291) in wild felid blood, spleen, and liver samples (1/44 *Leopardus pardalis*, 3/61 *Leopardus geoffroyi*, 1/66 *Panthera onca*, 1/3 *Herpailurus yagouaroundi*, and 3/32 *Leopardus wiedii*) (Supplementary material 2). Among the positive samples, the number of copies of a fragment of the *Bartonella* spp. *nuoG* gene per microliter could be estimated for seven of the nine samples (7/9) (Supplementary Material 2). The Efficiency, R^2^, Y-intercept and slope values of the qPCR assays varied between 99.2 and 104.9%, 0.980 to 0.998, 37.142 to 38.238 and − 3.209 to -3.342, respectively, according to MIQE recommendations (Bustin et al. 2009).

Among the nine qPCR-positive samples, amplification was obtained for the *gltA* gene in 2/9 (22.2%) samples, *nuoG* in 3/9 (33.3%), *ftsZ* in 1/9 (11.1%), and *rpoB* in 1/9 (11.1%). Of the PCR products submitted to Sanger sequencing, only three yielded high-quality readable sequences, corresponding to two *gltA* sequences obtained from *L. geoffroyi* and *L. wiedii* and one *ftsZ* sequence obtained from *L. geoffroyi* (Table 1).

**Table 1:** BLASTn analysis of partial sequences of the *gltA* and *ftsZ* genes obtained from spleen and liver samples of *Leopardus geoffroyi *and* Leopardus wiedii*

Sample ID (blood)	GenBank accession number	Host	Molecular marker	Size (bp)	Result	Host	QC	E value	Identity	Location	Accession number
DL01 (ID 852)	PX527374	*Leopardus geoffroyi*	*gltA*	379	*Bartonella* sp.	*Hylaeamys* *perenensis*	100%	0.0	98.15%	Peru	*KF021602.1*
DL01 (ID 1392)	PZ600596	*Leopardus wiedii*	*gltA*	365	*Bartonella henselae*	*Felis catus*	100%	0.0	100%	China	*MK729772.1*
DL06 (ID 852)	PZ600597	*Leopardus geoffroyi*	*ftsZ*	570	*Bartonella* sp.	Wild rodent	96%	0.0	99.82%	Brazil	*KX036245.1*
					*Bartonella machadoae*	*Thrichomys fosteri*	100%	0.0	96.84%	Brazil	*CP087114.1*

**Fig. 2:**
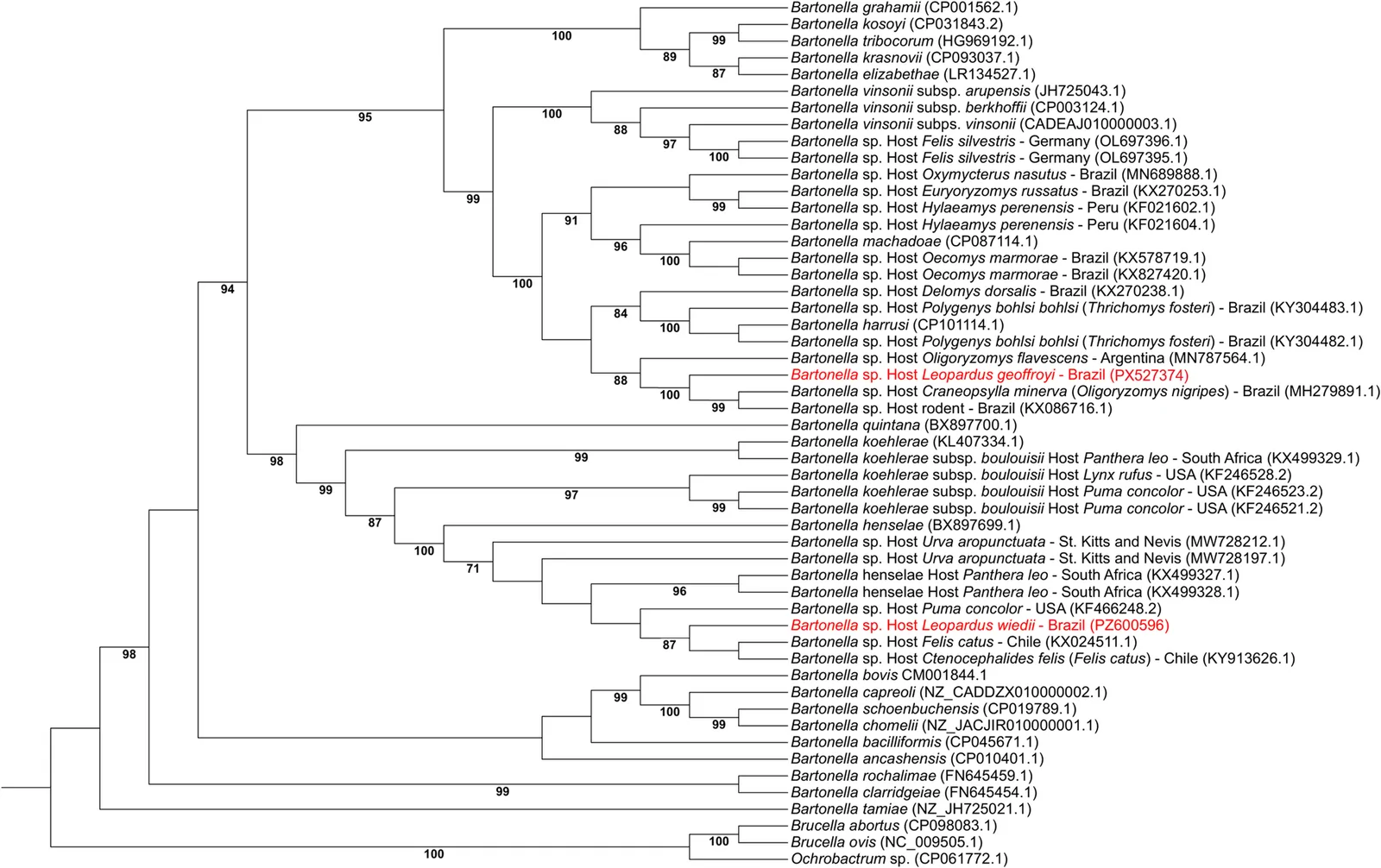
Phylogenetic tree inferred by the maximum likelihood (ML) method using a 369 bp alignment of the *gltA* gene using TIM3 + F + I+G4 as the evolutionary model. Sequences obtained in the present study, and their respective accession numbers are highlighted in red. *Brucella abortus*, *Brucella ovis* and *Ochrobactrum* sp. were used as outgroups. Only bootstrap values higher than 70 were shown. Genbank accession numbers are provided in parenthesis

The BLASTn analysis demonstrated that the *gltA* sequences obtained one from *L. wiedii* (PZ600596) and another one from *L. geoffroyi* (PX527374) had 100% identity with a *Bartonella henselae* sequence previously detected in *Felis catus* from China (MK729772.1) and 98.15% with *Bartonella* sp. sequence previously detected in *Hylaemys perenensis* from Peru (KF021602.1), respectively (Table 1). In the Maximum Likelihood analysis of the *gltA* region, the 372 bp *Bartonella* sp. sequence obtained from *L. geoffroyi* (PX527374) was positioned in the same sub-clade as *Bartonella* sp. sequences previously detected in wild rodents (KX086716.1; MN787564) and their associated ectoparasites (*Craneopsylla minerva* collected from an *Oligoryzomys nigripes*; MH279891.1) from Brazil. This sub-clade was closely related to a sub-clade containing *Bartonella harrusi* and *Bartonella* sp. sequences detected in rodents (*Delomys dorsalis*) (KX270238) and *Thrichomys fosteri*-associated *Polygenys bohlsi bohlsi* fleas (KY304482; KY304483). The 386 bp *Bartonella* sp. *gltA* sequence detected in *L. wiedii* (PZ600596) was positioned in the same sub-clade as *Bartonella* sp. sequences previously detected in domestic cats and their associated *Ctenocephalides felis felis* fleas from Chile (KX024511; KY913626) and *Bartonella* sp. previously detected in a *Puma concolor* from the USA (KF466248). This sub-clade was positioned in the same clade as *B. henselae* (Fig. 2).

**Fig. 3:**
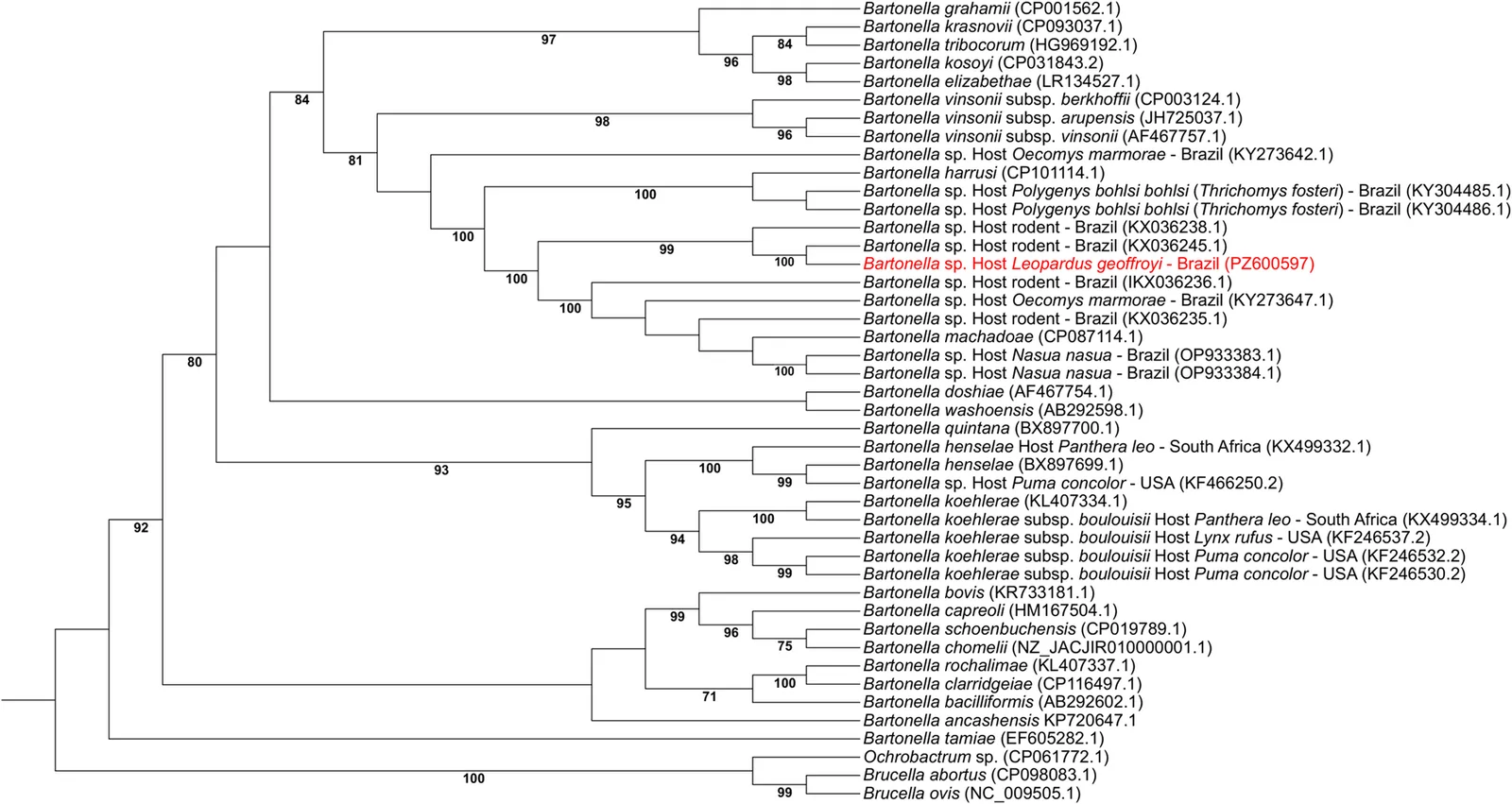
Phylogenetic tree inferred by the maximum likelihood (ML) method using a 570 bp alignment of the *ftsZ* gene using HKY + F+I+G4 as the evolutionary model. The sequence obtained in the present study, and its respective accession number are highlighted in red. *Brucella abortus*, *Brucella ovis* and *Ochrobactrum* sp. were used as outgroups. Only bootstrap values higher than 70 were shown

The BLASTn analysis demonstrated that the *Bartonella* sp. *ftsZ* sequence detected in *L. geoffroyi* (PZ600597) had an identity value of 99.82% with *Bartonella* sp. previously detected in a wild rodent from Brazil (KX036245.1) (Table 1) and 96.84% with *Bartonella machadoae* previously detected in *Thrichomys fosteri* from Brazil (CP087114.1). In the Maximum Likelihood analysis of the *ftsZ* region, the 581 bp *Bartonella* sp. sequence obtained from *L. geoffroyi* (PZ500697) was positioned in the same sub-clade as *Bartonella* sp. sequences previously detected in rodents from Brazil (KX036238; KX036245). These sequences were closely related to other *Bartonella* sp. sequences previously detected in rodents and their associated ectoparasites from Brazil, *Bartonella harrusi* and *B. machadoae* (Fig. 3).

## Discussion

Despite the well-known relationship between *Bartonella* spp. and domestic cats, to date, there is still a paucity of reports on *Bartonella* spp. in wild felids, mainly in South America. Herein, we report molecular evidence of the circulation of *Bartonella* spp. in 3.09% (9/291) of free-living wild felids from municipalities in the states of Bahia (northeastern), Minas Gerais (southeastern), and Rio Grande do Sul (southern).

Wild felids are susceptible to many of the same pathogens that affect domestic cats, several of which can be transmitted both within and between species, especially in ecosystems increasingly threatened by the diminishing separation between domestic and wild mammals (Obiegala et al. 2024). Indeed, previous studies conducted in the southern and southeastern regions of Brazil have detected *B. henselae* and *B. koehlerae* in wild felids (Guimarães et al. 2010; Filoni et al. 2012; Souza et al. 2021). Interestingly, the present study detected not only *B. henselae*, a species well recognized whose main reservoirs are represented by cats, but also *Bartonella* species associated with wild small mammals and associated ectoparasites were identified. In a preliminary analysis, the molecular detection of wild rodents and marsupials-associated *Bartonella* spp. in blood samples from *L. geoffroyi* may suggest that predatory behavior possibly increases contact not only with prey but also with their ectoparasites, facilitating pathogen exchange. Similarly, *Bartonella taylorii*, a rodent-associated *Bartonella* species, has been reported in wild felid species across Europe, including *Felis silvestris* in Germany (Obiegala et al. 2024) and *Lynx lynx* in the Czech Republic (Danek et al. 2023).

In the phylogenetic analysis based on the *gltA* gene, the sequences obtained in the present study were positioned within two distinct groups. The sequence obtained from *L. wiedii* was positioned within a group containing *B. henselae* sequences, whereas the sequence obtained from *L. geoffroyi* was positioned within a group containing *Bartonella* sequences previously detected in the rodent-associated *C. minerva* fleas from southern Brazil (Schott et al. 2020) and *P. bohlsi bohlsi* from the Brazilian Midwest (de Sousa et al. 2018). However, the relatively short *gltA* fragments obtained in the present study limited the resolution of phylogenetic inference, and the observed clustering should therefore be interpreted cautiously. Both *C. minerva* and *P. bohlsi bohlsi* primarily parasitize wild rodents and marsupials, which are common prey of wild felids in Brazil (de Moraes et al. 2003). Similarly, the phylogenetic analysis based on the *ftsZ* gene showed that the sequence detected in *L. geoffroyi* clustered with *Bartonella* sequences previously detected in rodents and associated ectoparasites. These findings suggest that wild felids may act as accidental hosts of *Bartonella* species associated with wild rodents and marsupials and their respective ectoparasites, possibly as a consequence of predation (Danek et al. 2023; Obiegala et al. 2024).

Herein, a *ftsZ* sequence showing identity < 97% with *B. machadoae* was detected in *L. geoffroyi* from southern Brazil, suggesting that wild felids may be exposed to *Bartonella* spp. associated with wild rodents. However, considering that rodent-associated *B. machadoae* and marsupial-associated *B. harrusi* are phylogenetically closely related, distinction between these two *Bartonella* species cannot be feasible using cut-offs previously established for several molecular markers (do Amaral et al. 2022). Therefore, the real identity of the two *Bartonella* genotypes detected herein remain unknown. Nonetheless, the findings presented herein show, for the first time, the circulation of small mammals-associated *Bartonella* spp. in wild felids from southern Brazil.

Considering that previous studies have shown that the combination of pre-enrichment liquid culture (e.g., BAPGM, *Bartonella* AlphaProteobacteria Growth Medium) and isolation in blood/chocolate agar with qPCR assays can enhance the detection of *Bartonella* spp. (Dias et al. 2023), the molecular occurrence reported herein might be underestimated. Other approaches, including digital PCR (dPCR) (Maggi et al. 2020), the combination of PCR and serological assays, and microscopic examination, may also contribute to improving the detection of *Bartonella* spp. in biological samples and should be considered in future investigations. Future studies aiming at performing isolation and Whole Genome Sequencing of Neotropical wild-felids-associated *Bartonella* isolates are much needed to shed light on the diversity of bartonellae in these mammals.

The impact of *Bartonella* spp. on wild felid populations and associated zoonotic risks remain unknown, as does the potential role of these animals in maintaining and disseminating these pathogens. The lack of identification of *Bartonella* spp., usually found in domestic cats in *L. geoffroyi* suggests minimal interaction of the sampled animals with domestic cats, supporting a sylvatic pathogen life cycle in wild felids. The set of data in this study expands our knowledge of this emerging pathogens and highlights the need for further research on its impact on wildlife.

In a nutshell, these findings reinforce that besides harboring *Bartonella* species associated with cats, wild felids also may act as accidental hosts for *Bartonella* species associated with wild small mammals, possibly as a consequence of predation.

## Supplementary Information

Below is the link to the electronic supplementary material.

Supplementary Material 1

Supplementary Material 2

Supplementary Material 3

## Data Availability

The sequences generated during the presents study were deposited in the NCBI Genbank (https://www.ncbi.nlm.nih.go v/genbank/). Sequences can be accessed by the following accession numbers: gltA (PZ600596, PX527374) and ftsZ (PZ600597).
