# Supplementary material for: Molecular evidence of Bartonella spp. in wild felids from Brazil
Source: Vet Res Commun. 2026 Oct 17;51(1):7. doi: 10.1007/s11259-026-11579-5 (PMC13676676; doi:10.1007/s11259-026-11579-5)
Supplement: Supplementary file 1 [file 11259_2026_11579_MOESM1_ESM.docx]

**Supplementary material_1**

Description of the PCR assays used in this study molecular screening and characterization for *Bartonella* spp..

| Molecular Marker | Primers/Probe | Thermal conditions | Amplicon size (bp) | Reference |
| --- | --- | --- | --- | --- |
| *nuoG*  (qPCR) | **F**  (5’-CAATCTTCTTTTGCTTCACC- 3’)  **R**  (5’-TCAGGGCTTTATGTGAATAC -3’)  **Probe**  FAM-5’- TYGTCATTTGAACACG3’[BHQ2a-Q]3 | 95ºC for 3min; 40 cycles of 95ºC for 10s, 52.8ºC for 30s | 83 | (André et al., 2015) |
| *gltA* | **BhCS.781p (gltA F)**  (5’ GGGGACCAGCTCATGGTGG- 3’)  **BhCS.1137n (gltA R)**  (5’ AATGCAAAAAGAACAGTAAACA- 3’) | 95ºC for 10min; 35 cycles of 95ºC for 20s, 51ºC for 30s and 72ºC for 2min; 72ºC for 3min | 380-400 | (Norman et al., 1995) |
|  | **CS443f**  (5’ -GCTATGTCTGCATTCTATCA- 3’)  **CS1210r**  (5’ -GATCYTCAATCATTTCTTTCCA- 3’) | 94ºC for 2min; 45 cycles of 94ºC for 30s, 48ºC for 1min and 72ºC for 1min; 72ºC for 7min | 767 | (Billeter et al., 2011) |
| *ftsZ* | **ftsZ F**  (5’ -CATATGGTTTTCATTACTGCYGGTATGG- 3′)  **ftsZ R**  (5’ -TTCTTCGCGAATACGATTAGCAGCTTC- 3′) | 94ºC for 2min; 40 cycles of 94ºC for 2min, 61ºC for 45s and 72ºC for 45s; 72ºC for 7min | 515 | (Paziewska et al., 2011) |
| *groEL* | **GroEL F**  (5′ -GGAAAAAGTGGGCAATGAAG- 3′)  **GroEL R**  (5′ -TCCTTTAACGGTCAACGCATT- 3′) | 94ºC for 2min; 40 cycles of 94ºC for 2min, 47ºC for 45s and 72ºC for 45s; 72ºC for 7min | 752 | (Paziewska et al., 2011) |
| *nuoG*  (conventional) | **nuoG F**  (5’ -GGCGTGATTGTTCTCGTTA- 3’)  **nuoG R**  (5’ -CACGACCACGGCTATCAAT -3’) | 94ºC for 5min; 35 cycles of 94ºC for 30s, 53ºC for 30s and 72ºC for 5min; 72ºC for 5min | 346 | (Colborn et al., 2010) |
| *Pap31* | **165s**  (5’ -GACTTCTGTTATCGCTTTGATTT- 3’)  **688as**  (5’ -CACCACCAGCAAMATAAGGCAT- 3’) | 95ºC for 5min; 45 cycles of 94ºC for 30s, 56ºC for 30s and 72ºC for 45s; 72ºC for 5min | 564 | (Maggi & Breitschwerdt, 2005^a^) |
| *rpoB* | **1400F**  (5’ -CGCATTGGCTTACTTCGTATG- 3’)  **2300R**  (5’ -GTAGACTGATTAGAACGCTG- 3’) | 94ºC for 2min; 35 cycles of 94ºC for 30s, 53ºC for 30s and 72ºC for 1min; 72ºC for 2min | 825 | (Renesto et al., 2001) |
| *ribC* | **Barton-1**  (5’ -TAACCGATATTGGTTGTGTTGAAG- 3’)  **Barton-2**  (5’ -TAAAGCTAGAAAGTCTGGCAACATAACG- 3’) | 95ºC for 10min; 37 cycles of 95ºC for 1min, 51ºC for 1min and 72ºC for 1min; 72ºC for 3min | 585-588 | (Johnson et al., 2003) |
