# Supplementary material for: Molecular evidence of Bartonella spp. in wild felids from Brazil
Source: Vet Res Commun. 2026 Oct 17;51(1):7. doi: 10.1007/s11259-026-11579-5 (PMC13676676; doi:10.1007/s11259-026-11579-5)
Supplement: Supplementary file 2 [file 11259_2026_11579_MOESM2_ESM.docx]

**Supplementary material_2**

Identification, type and quantification of positive samples from wild felids submitted to qPCR assays for *Bartonella* spp. based on the *nuoG* gene.

| Wild felines | Sample identification – host specie | Sample type | Sample origen | Quantification of positive samples (qPCR for *Bartonella* spp. based on the *nuoG* gene) |
| --- | --- | --- | --- | --- |
|  | 1407 – *Leopardus wiedii* | blood | Quevedos (RS) | 2,19 x 10^0^ |
|  | 1292 – *Herpailurus yagouaroundi* | blood | Paracatu (MG) | 6,77 x 10^-1^ |
|  | 1698 – *Leopardus wiedii* | spleen | Muçum (RS) | 8,62 x 10^-1^ |
|  | 351 – *Panthera onca* | blood | Cocos (BA) | 5,66 x 10^1^ |
|  | 1474 – *Leopardus pardalis* | blood | Paracatu (MG) | 5,98 x 10^-1^ |
|  | 1986 – *Leopardus geoffroyi* | blood | Dom Pedrito (RS) | 3,85 x 10^1^ |
|  | 2508 – *Leopardus geoffroyi* | spleen | Bagé (RS) | 6,76 x 10^3^ |
|  | 852 – *Leopardus geoffroyi* | liver | Cachoeira do Sul (RS) | ** |
|  | 1392 – *Leopardus wiedii* | spleen | Dois Irmãos (RS) | ** |

**Positive sample not quantifiable probably due to the Monte Carlo effect.
